# Lack of serologic evidence of orthoflavivirus infection in dogs with meningoencephalitis of unknown origin and steroid-responsive meningitis-arteritis in the Netherlands

**DOI:** 10.1177/10406387251340619

**Published:** 2025-05-21

**Authors:** Koen M. Santifort, Kiki Streng, Niklas Bergknut, Iris Van Soens, Marta Plonek, Wim H. M. van der Poel

**Affiliations:** IVC Evidensia Small Animal Referral Hospital Arnhem, Arnhem, The Netherlands; IVC Evidensia Small Animal Referral Hospital Hart van Brabant, Waalwijk, The Netherlands; Infectious Disease Epidemiology, Wageningen University & Research, Wageningen, The Netherlands; IVC Evidensia Small Animal Referral Hospital Hart van Brabant, Waalwijk, The Netherlands; IVC Evidensia Small Animal Referral Hospital Hart van Brabant, Waalwijk, The Netherlands; IVC Evidensia Small Animal Referral Hospital Arnhem, Arnhem, The Netherlands; Infectious Disease Epidemiology, Wageningen University & Research, Wageningen, The Netherlands; Wageningen Bioveterinary Research, Lelystad, The Netherlands

**Keywords:** canine, meningoencephalitis of unknown etiology, Netherlands, Orthoflavivirus, serology

## Abstract

The pathogenesis of meningoencephalomyelitis of unknown origin (MUO) and steroid-responsive meningitis-arteritis (SRMA) in dogs remains enigmatic. Numerous studies have attempted and failed to identify (viral) pathogens in samples from MUO- or SRMA-diagnosed dogs. Orthoflavivirus-associated meningoencephalitis or meningoencephalomyelitis has been diagnosed in dogs in several European countries. We investigated serologic evidence for orthoflavivirus infection in dogs with clinical diagnoses of MUO or SRMA in the Netherlands. Twelve dogs with a clinical diagnosis of MUO based on signalment, neurologic examination, MRI studies, CSF analysis, and response to treatment were included in the study (age range: 1–11 y; 4 females, 8 males; weight range: 8–44 kg). Serum samples from all 12 dogs tested negative in a commercial competitive ELISA and virus neutralization tests for West Nile virus, Usutu virus, and tick-borne encephalitis virus. We did not find serologic evidence of orthoflavivirus infection in dogs with MUO or SRMA in the Netherlands.

The terms *meningoencephalomyelitis of unknown origin* (MUO) and *steroid-responsive meningitis-arteritis* (SRMA) refer to inflammatory disorders of the CNS. MUO variants are characterized and classified histologically as necrotizing meningoencephalitis, necrotizing leukoencephalitis, and granulomatous meningoencephalitis.^8^ MUO can account for ~50% of CNS inflammatory disorders in dogs.^11^ To date, it is deemed most likely that MUO and SRMA are autoimmune disorders.^2,3,8^ Indeed, autoantibodies against neuronal and glial antigens have been identified in some cases.^2,3^ Nevertheless, it is possible that, in some patients, MUO has an infectious cause that is not identified by clinical or histologic tests. Orthoflaviviruses such as tick-borne encephalitis virus (TBEV; *Flaviviridae*, *Orthoflavivirus encephalitidis*) and West Nile virus (WNV; *Flaviviridae*, *Orthoflavivirus nilense*) have caused meningoencephalitis in human patients in the Netherlands since ~2015.^19,23^ Recognizing the emerging relevance of orthoflaviviruses in clinical veterinary medicine,^9,10,12,13,15,16,18,20,21^ we investigated serologic evidence for orthoflavivirus infection in dogs with MUO in the Netherlands.

Sampling of the animals in our study was performed under legislation (license AVD40100202114384) of the Dutch Central Authority for Scientific Procedures on Animals (CCD), and the experimental plan was approved by the Animal Welfare Body of Wageningen University and Research prior to the start of the sampling. For our prospective study, we included dogs with a clinical diagnosis of MUO or SRMA based on signalment, neurologic examination, and either or both MRI studies, and CSF analysis between May 2021 and May 2022. This subset of dogs was part of a larger serosurvey set up to investigate the seroprevalence of orthoflaviviruses in horses and dogs in the Netherlands.^22^ Serum samples were screened for antibodies against orthoflaviviruses by a commercial multi-species WNV competitive ELISA (ID Screen; IDvet). Additionally, virus neutralization tests (VNTs) were performed for WNV, Usutu virus (USUV; *Flaviviridae*, *Orthoflavivirus usutuense*), and TBEV as described previously.^22^

We included 12 dogs (age range: 1–11 y; 4 females, 8 males; weight range: 8–44 kg) in our study. All dogs spent 0–6 h outdoors per day. Of these 12 dogs, 8 were diagnosed with MUO and the remaining 4 with SRMA. Four dogs recovered fully, 3 recovered partially, and 5 dogs were euthanized. All serum samples tested negative for the presence of orthoflavivirus antibodies (Table 1). Seven dogs were tested for canine distemper virus (CDV) among other pathogens, for which all results were negative.

**Table 1.** Signalment and case information of dogs with a clinical diagnosis of meningoencephalitis of unknown origin, from which serum samples were obtained and included in the study.

Case	Age, y	Sex	Breed	Weight, kg	Housing area	Insect repellent used during vector season	Tick*	ELISA S/N value, %	
1	4	M	Cane Corso	44	Urban	Y	N	106	
2	1	CM	Staffordshire Terrier	28	Urban	Y	Y	65.6	
3	4	CF	Mixed	11	Urban	Y	N	91.9	
4	1	M	Friese Stabij	21	Urban	N	N	79.7	
5	1	M	Golden Retriever	32	Urban	Y	N	84.7	
6	11	M	Mixed	22	Urban	N	N	78.5	
7	7	M	Mixed	8	Urban	N	N	77	
8	1	CF	Labrador Retriever	31	Agricultural	Y	Y	68.1	
9	1	M	Beagle	14	Urban	N	N	95	
10	1	F	Cavalier King Charles Spaniel	12	Urban	N	N	67.5	
11	4	F	Cairn Terrier	8	Urban	N	N	69.5	
12	4	M	Vizsla	29	Urban	N	N	91.2	
	Duration of clinical signs, days before serum sampling	Clinical signs	Neuroanatomical localization	MRI abnormalities	CSF	Other tests	Clinical diagnosis	Response to treatment†	Outcome
1	7	Mild obtundation, head and body turn to the right, video of contralateral head and body turn, proprioceptive deficits, positional ventral strabismus right eye	Multifocal intracranial	Multifocal intra-axial	TNCC 0.004 × 10^9^/L, protein 0.201 g/L, cytology: lymphocytes	Serology: *T. gondii* IgM < 1:16 and IgG < 1:32 (IFT), *N. caninum* (Ab) < 1:160 (IFT)	MUO	Partial, added cyclosporin 5 mg/kg q12h, incomplete recovery and persistent neurologic deficits	Euthanasia
2	5	Abnormal behavior, head turn and circling left, proprioceptive deficits, abnormal pupillary light reflexes	Multifocal intracranial	Multifocal intra-axial	TNCC 0.68 × 10^9^/L, protein 0.40 g/L, cytology: lymphocytic pleocytosis	PCR for *Bartonella* spp., CDV, *Neospora* spp., *T. gondii* on CSF, all negative	MUO	Partial, added cyclosporin 5 mg/kg q12h	Altered personality remained
3	1	Abnormal behavior, epileptic seizures, ataxia, proprioceptive deficits, menace responses absent, strabismus left eye	Multifocal intracranial	Multifocal intra-axial	TNCC 0.01 × 10^9^/L, cytology: lymphocytic pleocytosis	PCR for *Bartonella* spp., CDV, *Neospora* spp., *T. gondii* on CSF, all negative	MUO	Good response initially, recurrence of seizures and neurological deficits after 3 mo	Euthanasia
4	10	Tetraparesis, non-ambulatory, spasticity, cervical hyperesthesia	C1-5 myelopathy	Multifocal cervical spinal cord	TNCC 0.14 × 10^9^/L, protein 1.95 g/L, cytology: lymphocytic pleocytosis	PCR for *Bartonella* spp., CDV, *Neospora* spp., *T. gondii* on CSF, all negative	MUO	Partial, added ciclosporin 5 mg/kg q12h	Euthanasia
5	14	Fever, tetraparesis, ataxia, cervical hyperesthesia, proprioceptive deficits	C1-5 myelopathy	Multifocal cervical spinal cord	TNCC 0.12 × 10^9^/L, protein 7.72 g/L, cytology: neutrophilic pleocytosis	PCR for *Bartonella* spp., CDV, *Neospora* spp., *T. gondii* on CSF, all negative	SRMA	Complete response	Complete recovery
6	2	Fever, cervical hyperesthesia	Hyperesthesia, no neurological deficits	—	TNCC 0.05 × 10^9^/L, protein 0.21 g/L, cytology: neutrophilic pleocytosis	—	SRMA	Complete response	Complete recovery
7	30+	Abnormal behavior, epileptic seizures, ataxia, proprioceptive deficits, menace response absent on the right, palpebral reflex absent left	Multifocal intracranial	Multifocal intra-axial	TNCC 0.01 × 10^9^/L, protein 1.41 g/L, cytology: lymphocytic pleocytosis	—	MUO	Partial response	Euthanasia
8	1	Cerebellar ataxia, wide-based stance, intention tremor	Cerebellum	Cerebellum intra-axial	TNCC 0.01 × 10^9^/L, protein 0.31 g/L, cytology: lymphocytic pleocytosis	PCR for *Bartonella* spp., CDV, *Neospora* spp., *T. gondii* on CSF, all negative	MUO	Complete response	Complete recovery
9	7	Fever, abnormal behavior, ataxia, epileptic seizures, obtundation, tremors	Multifocal intracranial	Multifocal intra-axial	TNCC 0.01 × 10^9^/L, cytology: lymphocytic pleocytosis	Serology: *T. gondii* IgM < 1:16 and IgG < 1:32 (IFT), *N. caninum* (Ab) < 1:160 (IFT)	MUO	Partial, added ciclosporin 5 mg/kg q12h	Partial recovery, stable, abnormal behavior and ataxia persists
10	5	Fever, obtundation, cervical hyperesthesia	Multifocal	—	TNCC 0.24 × 10^9^/L, cytology: neutrophilic pleocytosis	CSF bacterial aerobic and anaerobic culture-negative	SRMA	Complete response, recurrence of signs after 5 mo, restarted treatment as before	Complete recovery
11	4	Myoclonic epileptiform seizures, spastic tetraparesis, proprioceptive deficits, hypermetric ataxia, absent menace responses both eyes	Multifocal	Multifocal intra-axial	TNCC 0.50 × 10^9^/L, cytology: lymphocytic pleocytosis	Serology: *T. gondii* IgM < 1:16 and IgG < 1:32 (IFT), *N. caninum* (Ab) < 1:160 (IFT) PCR for *Bartonella* spp., CDV, *Neospora* spp., *T. gondii* on CSF, all negative	MUO	Partial response, recurrent epileptiform seizures despite anti-epileptic medication	Euthanasia
12	8	Obtundation, tetraparesis, ataxia, truncal sway, proprioceptive deficits, menace response absent right eye, vertical positional nystagmus, decreased gag reflex	Multifocal intracranial	Multifocal intra-axial	TNCC 0.008 × 10^9^/L, protein 4.33 g/L, cytology: mononuclear pleocytosis	Serology: *T. gondii* IgM < 1:16 and IgG < 1:32 (IFT), *N. caninum* (Ab) < 1:160 (IFT) PCR for *Bartonella* spp., CDV, *Neospora* spp., *T. gondii* on CSF, all negative	MUO	Partial response	Partial recovery, persistent mild obtundation

Ab = antibody; CDV = canine distemper virus; CM = castrated male; CSF = cerebrospinal fluid (RI: <0.003 × 10^9^/L); F = female; IFT = indirect fluorescent antibody test; M = male; MUO = meningoencephalomyelitis of unknown origin; N = no; *N. caninum* = *Neospora caninum*; SF = spayed female; SRMA = steroid-responsive meningitis-arteritis; TNCC = total nucleated cell count; *T. gondii* = *Toxoplasma gondii*; Y = yes; — = MRI not performed.

* Tick removed by owner in 12 mo prior to sampling.

† All dogs were treated with immunosuppressive dosages of prednisolone (1–2 mg/kg q12h) followed by a tapering scheme (possibly with the addition of other treatments not specified, such as anti-epileptic medications).

Numerous studies have attempted but largely failed to identify evidence for the presence of, or role for, various pathogens, such as astroviruses, bornaviruses, CDV, *Toxoplasma gondii*, and *Borrelia burgdorferi*, in samples of dogs with a clinical diagnosis of MUO or SRMA.^2,3,7^ When an infectious cause is identified, the term MUO or SRMA is discarded. Orthoflaviviruses may cause meningoencephalomyelitis in dogs as well as other mammals, including humans.^1,6,12^ The *Orthoflavivirus* genus (positive-strand RNA viruses—arthropod vector-borne) includes WNV, USUV, TBEV, dengue virus, and yellow fever virus, among others.^14^ Dog ownership has been identified as a risk factor for TBE in humans.^17^ Ticks, such as *Ixodes ricinus*, are involved in the infection of dogs with TBEV.^4,21^ Diagnosis of TBE has been reached in other studies of dogs in Europe by identification of antibodies in serum and CSF and/or testing for viral RNA.^1,4,13^ Even though experimental WNV infections in dogs did not result in clinical signs,^5^ clinical WNV infections have been reported in which animals had signs of encephalitis and myocarditis, among other signs.^6^ We found no records of clinical cases of USUV in dogs in the literature.

The seroprevalence of orthoflaviviruses in dogs in Europe varies per country but may be up to 16.4% for TBEV and 55.5% for WNV.^10,16^ TBEV, USUV, and to a lesser extent WNV, are known to circulate in the Netherlands, as infections have been proven in both vectors and dead-end hosts.^9,18,20^ As we did not detect seropositive dogs, our study does not support a role for orthoflavivirus infection in dogs clinically diagnosed with MUO or SRMA in the Netherlands. Our results can, however, not exclude the possibility of orthoflaviviruses (or other unidentified pathogens) causing meningoencephalitis in dogs in the Netherlands, as we were not able to perform viral testing on relevant tissues and, for some of the dogs, the time to develop proper antibody responses may have been too short. Unfortunately, we were unable to perform additional sampling of the surviving animals and postmortem examination of dead animals.

As the search for pathogens involved in meningoencephalomyelitis in dogs continues, future studies should consider and possibly address several limitations of our study, including lack of concurrent antibody testing of CSF, small cohort size (12 dogs), imperfect sensitivity and specificity of the used serologic test, and timing of sampling (repeated sampling from the same animals was not performed to detect seroconversion). We encourage histologic confirmation of clinical diagnoses of MUO in future cases.
